# SepIVA has a function in activating the mycobacterial divisome, which is inhibited during DNA damage

**DOI:** 10.1128/mbio.01985-26

**Published:** 2026-09-22

**Authors:** Anusuya Nepal, Gwyneth Untalan, Cara C. Boutte

**Affiliations:** 1 Department of Biology, University of Texas Arlington 171766 , Arlington, Texas, USA; Indiana University Bloomington, Bloomington, Indiana, USA

**Keywords:** cell division, mycobacteria, DNA damage checkpoints, divisome assembly

## Abstract

Bacterial cell division is a tightly regulated process that is carried out by a complex of proteins called the divisome. These proteins are assembled in a defined sequential order. Upon assembly, the complex is allosterically activated, which stimulates septal peptidoglycan synthesis at the nascent septum. Bacteria inhibit division during DNA damage by blocking either divisome assembly or activation. While the regulation of cell division is known to be important during *Mycobacterium tuberculosis* infection, little is known about mycobacterial mechanisms of divisome function and regulation in DNA damage. By using *Mycobacterium smegmatis* as a model organism, we find here that divisome factor SepIVA is involved in septum initiation and that it is recruited to the mid-cell by FtsQ, but it is not a recruitment factor itself. We find that a *sepIVA* loss-of-function defect can be suppressed by overexpression of *ftsW*, supporting a role for SepIVA in activation of peptidoglycan synthesis within the divisome. When cell division is inhibited during DNA damage, we find that SepIVA is delocalized from the division site, while the septal localization of FtsZ, FtsQ, and FtsW is not impacted. We also find that the interaction between FtsQ and SepIVA is inhibited during DNA damage. Our results suggest that SepIVA is involved in the activation of septal synthesis during normal growth and show that the signaling to inhibit cell division during DNA damage involves inhibition of SepIVA’s interaction with FtsQ.

**IMPORTANCE:**

Cell division is critical for bacterial cells to propagate and cause infection. Despite its importance, division is a dangerous process as it requires building and subsequently hydrolyzing new cell wall material in a place where the chromosome typically resides. If cell division occurs before chromosome segregation is completed, or if cell wall metabolism is improperly regulated, then cell death results. The divisome is a protein complex that regulates cell division—coordinating it with the status of the chromosome and ensuring that the cell wall metabolic enzymes are carefully controlled. Many of the proteins in the divisome complex are highly conserved across bacterial phyla; however, the factor that stimulates the complex to activate and initiate cell wall synthesis is not widely conserved. Here, we study the activation of the divisome in *Mycobacterium smegmatis*, a model for cell physiology of *Mycobacterium tuberculosis*. We find evidence that SepIVA, a protein found only in Actinomycetota, promotes activation of peptidoglycan synthesis at the divisome in mycobacteria. We also show that the association of SepIVA with the divisome is blocked under DNA damage, when cell division is inhibited. These results provide a model for the regulation of cell division in mycobacteria in growth and stress and also provide insights into how bacteria with different types of septa may regulate division differently.

## INTRODUCTION

Cell division in bacteria is a highly controlled process. It is carried out by a multiprotein trans-membrane complex called the divisome (1–4). The complex includes cytoskeletal factors, transmembrane proteins, and peptidoglycan synthases and hydrolases (5, 6) that work together to ensure that cell division occurs at the correct time and place and that the integrity of the cell wall is maintained during the division process. Cell division is carefully regulated in response to nutrient and stress conditions (7–14).

Much of our understanding of bacterial cell division comes from *Escherichia coli*—using it as a model, we can divide essential divisome factors into functional categories. There are proteins required for septum formation and those that resolve the septum to allow separation of the daughter cells (15, 16). Among septal formation factors, we can separate out the enzymes FtsW and FtsI, which do the chemical work of building the septal peptidoglycan (17–20), from the regulators that control the activity of the enzymes. Among regulators of septum formation, there are three functional categories. First, membrane anchors such as ZipA and FtsA identify and establish the division site at the mid-cell (21–23). FtsA acts by anchoring FtsZ polymers to the inner membrane, and ZipA helps tether and stabilize the Z-ring at the membrane (23, 24). Second, recruitment and scaffolding factors promote assembly of the divisome. These include the cytoskeletal protein FtsZ, the FtsK DNA translocase (25), and the transmembrane factors FtsQ, FtsL, and FtsB (26). The third category of septal formation regulators are allosteric activators—in *E. coli*, FtsN’s association with the divisome triggers conformational changes in FtsA and FtsQLB that activate FtsWI (27–30).

Several components of the *E. coli* divisome are conserved in mycobacteria (31, 32), including FtsZ, FtsQ, FtsL, FtsB, FtsK, and FtsWI (32). SepF tethers FtsZ polymers to the cytoplasmic membrane at mid-cell, instead of FtsA/ZipA in *E. coli* (33–35). The recruitment ordering of FtsZ, FtsL, FtsQLB, and FtsWI appears to be similar to that in *E. coli* (32). The step of sensing the completion of assembly of the divisome and triggering activation of FtsWI is undescribed in mycobacteria, and there is no homolog of FtsN (36). Inter-divisome interactions may contribute to allosteric activation: mycobacterial FtsZ directly interacts with FtsW (37) and with FtsQ (38), though the function of these interactions is not clear yet.

SepIVA is a protein broadly conserved across Actinomycetales (32, 39); however, its function does not appear to be conserved. In *Mycobacterium smegmatis* (*Msmeg*), SepIVA is essential for division (32), while in *Streptomyces venezuelae*, it does not have a role in division (39). In *M. smegmatis*, SepIVA localizes to the septum and sub-polar regions (32, 40). The sub-polar localization of SepIVA appears to have a modest role in regulating polar growth, possibly through recruitment of MurG to the poles (40). Although the septal localization of SepIVA occurs only briefly during the cell cycle, its septal function is essential (32, 40). SepIVA interacts with conserved divisome protein FtsQ (32, 41), and depletion of SepIVA does not affect the formation of the FtsZ ring, indicating that it is not a membrane anchor or an early recruitment factor (32). Time-lapse microscopy of fluorescent protein fusions showed that SepIVA’s localization to the divisome occurs ~70% of the way through the cell cycle, whereas FtsZ localizes at 30%, and FtsQLB localizes ~60% of the way through the cell cycle, indicating that SepIVA is a late recruit to the divisome (32). SepIVA has no predicted enzymatic activity (32).

Cell division in bacteria must occur in coordination with DNA replication and integrity, and division is inhibited under DNA damage (42, 43). Cell division is halted at the steps of divisome assembly or activation to allow DNA repair while the cell is diploid (43). DNA damage activates the highly conserved SOS response in bacteria: this involves de-repression of DNA repair genes by the transcription factor LexA (44). In *E. coli*, SOS-dependent SulA blocks cell division (45) by inhibiting the assembly of FtsZ (46). In *Caulobacter crescentus*, two transmembrane proteins inhibit cell division through interaction with the division subcomplex FtsW/I/N (10, 47). In *Bacillus subtilis*, SOS-induced YneA interacts with and inhibits FtsW to block divisome function (48–50). Thus, different organisms inhibit different steps of the septum formation process during DNA damage.

In mycobacteria, there are two transcriptional responses to DNA damage: the canonical SOS-response (51–54) and the PafBC pathway (53, 55, 56), both of which activate transcription of DNA recombination and repair factors. ChiZ has been proposed as an SOS-activated cell division inhibitor; however, it has not been shown to be necessary for this function (57, 58). How other divisome components might be regulated during DNA damage has not been studied in mycobacteria.

In this work, we examined the assembly of the divisome under growth and DNA damage, with a focus on the role of SepIVA in *M. smegmatis*. We show that SepIVA is required for septum formation, not septal resolution. Then, we examined the dependency of localization of several essential divisome factors and found that SepIVA is recruited by FtsQ to the mid-cell, but it is not itself a recruitment factor for essential divisome factors. We also showed that overexpression of FtsW could suppress a SepIVA loss-of-function mutant, which provides support for the model that SepIVA contributes to activation of FtsWI, possibly through its interaction with FtsQ. Under DNA damage, we found that division is inhibited but that FtsZ, FtsQ, and FtsW are not delocalized from mid-cell, while SepIVA is delocalized, and it dissociates from FtsQ. Our results suggest that SepIVA is involved in activation of cell division during normal growth and that its interaction with FtsQ is inhibited during DNA damage to inhibit cell division.

## RESULTS

### SepIVA is required for septum formation

SepIVA is essential for cell division in mycobacteria (32, 59); however, its specific molecular function is not described. Septal cross-wall formation and septal resolution are temporally distinct stages of cell division in mycobacteria (60, 61). Depletion of essential septal formation proteins results in long cells with fewer septa (32), while depletion of septal resolution factors results in long chained filaments with numerous septa (62). To determine which of these stages SepIVA is required for, we used an *M. smegmatis* strain which expresses SepIVA-FLAG-DAS that can be proteolyzed upon induction of the protease adaptor SspB (32, 63), resulting in a marked reduction in SepIVA protein levels (Fig. 1). To visualize sites of active cell wall assembly following SepIVA depletion, we stained the cells with the fluorescent D-amino acid HADA, which incorporates into nascent and remodeled peptidoglycan at the poles and septa (64, 65). Under SepIVA depletion, we observed a significant increase in the distances between the cell poles, septa, and successive septa, compared with control cells, where SepIVA is at normal levels (Fig. 1). We did not observe a change in the total ratio of septa to cells (control: 0.31 ± 0.039; depleted: 0.34 ± 0.075), because the SepIVA-depleted cells were longer. This decreased frequency of septa per unit of cell length indicates that SepIVA is critical for septal cross-wall formation and is not a septal resolution factor.

**Fig 1:**
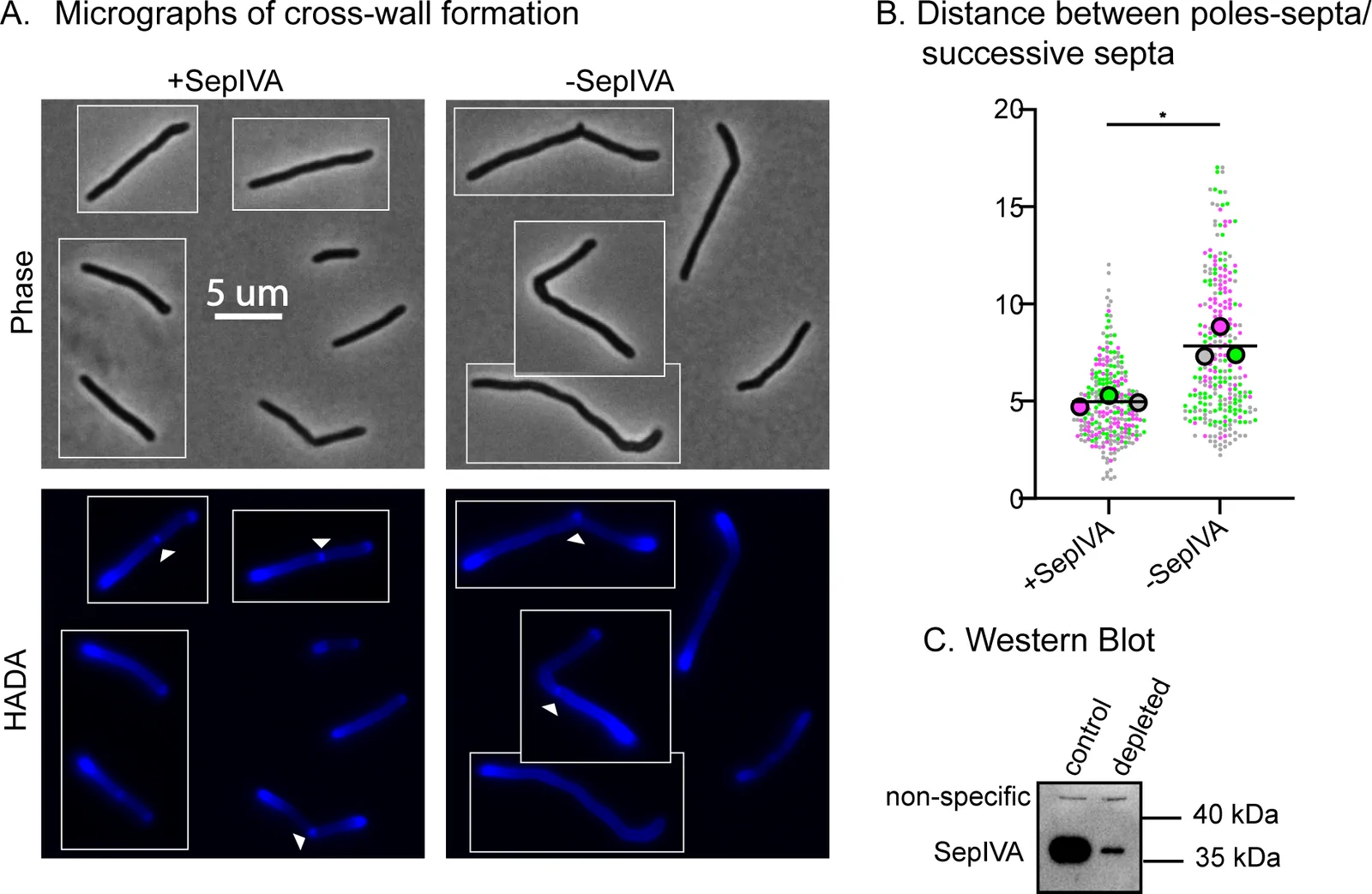
SepIVA is required for septum formation. (**A**) Micrographs of the SepIVA-DAS strain with stable SepIVA-DAS (left) and depletion of SepIVA-DAS by SspB expression (right) stained with HADA. The scale bar is 5 µm and applies to all images. Arrowheads represent the septa in the cells. (**B**) Distance between poles and septa or two successive septa of cells with septa and between two poles in cells without septa from panel **A**. A total of 250 cells over 3 biological replicates of SepIVA-DAS depleted strains and 230 cells over 3 biological replicates of control SepIVA-DAS strains were analyzed. The circles represent the mean distance of each replicate in the superplot. *, *P* = 0.0457. *P*-value was calculated by using a paired *t*-test. (**C**) α-FLAG Western blot of lysates from the SepIVA-DAS strain, without induction of the SspB proteolytic adaptor (control) and after 7–8 h of induction (depleted).

### SepIVA is recruited to the divisome site by FtsQ but does not recruit downstream divisome factors

In model species, essential non-enzymatic septum formation proteins are involved either in (i) membrane anchoring of the Z-ring, (ii) divisome complex assembly (16, 66–68), or (iii) allosteric activation of the cell division enzymes (27, 28, 69). We previously showed that SepIVA is not required for the septal localization of FtsZ, so it is not a membrane anchoring factor (32). Next, we tested if SepIVA has a role in divisome assembly. We hypothesized that SepIVA would be recruited to the divisome adjacent to FtsQ because SepIVA and FtsQ have been reported to interact (32, 41). To determine whether FtsQ recruits SepIVA in the divisome, we made an *M. smegmatis* strain, Ptet_OFF_::*ftsQ*/mCherry-SepIVA, which allows transcriptional depletion of *ftsQ* and localization of SepIVA. In the control, mCherry-SepIVA is distributed mostly at septal and sub-polar regions as previously described (32, 40) (Fig. 2A), whereas depletion of *ftsQ* results in decreased localization of SepIVA at the mid-cell (Fig. 2A and B; Fig. S2). We conclude that SepIVA depends on FtsQ for its recruitment to the mid-cell and is therefore a late recruit to the divisome.

**Fig 2:**
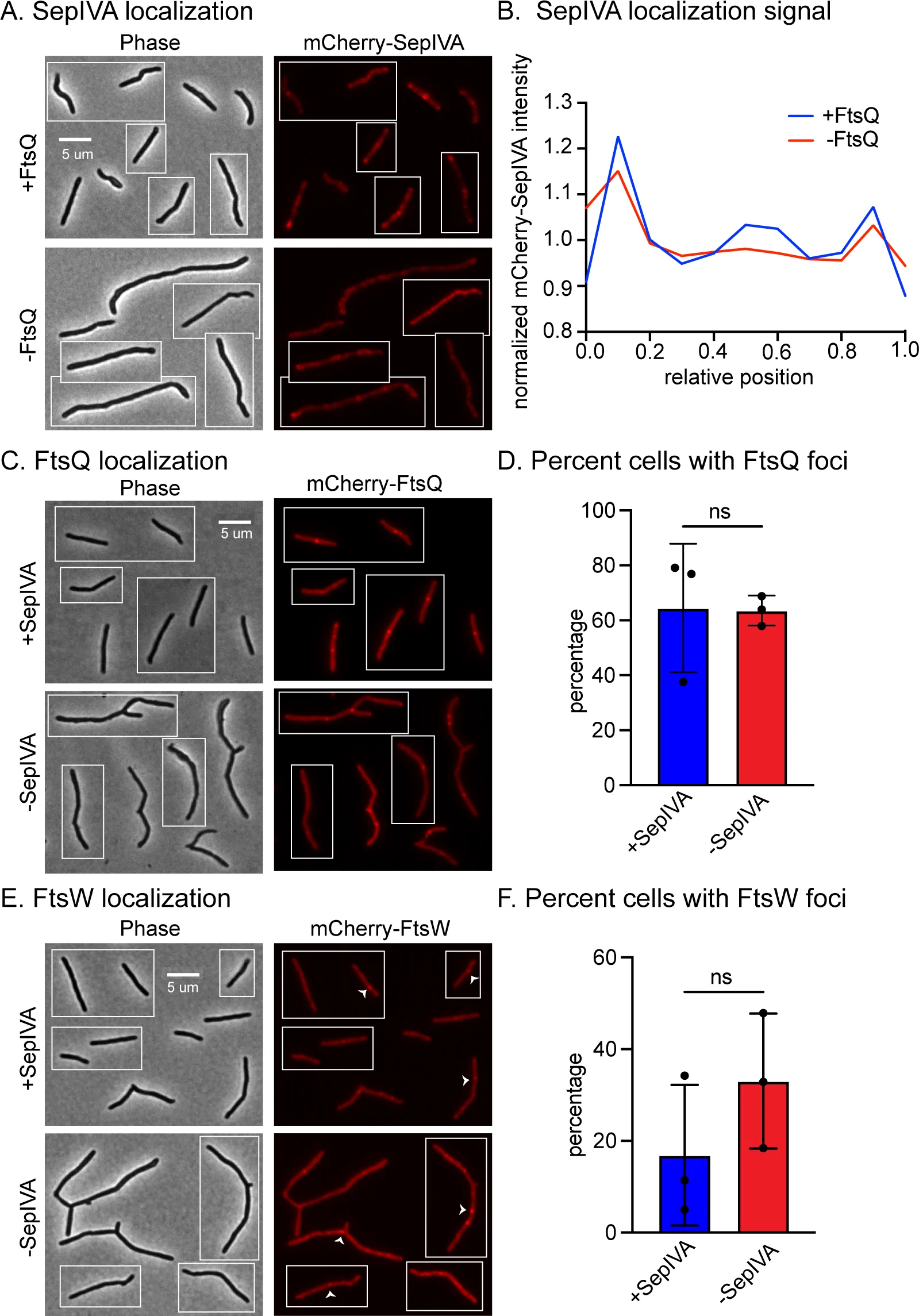
SepIVA is recruited to the divisome by FtsQ but does not recruit downstream divisome factors. (**A**) Micrographs of Ptet_OFF_::*ftsQ* strains expressing mCherry-SepIVA in the induced (top) and in transcriptional depletion of *ftsQ* (bottom). The scale bar is 5 µm and applies to all images in the panel. (**B**) Mean normalized intensity profile of mCherry-SepIVA signal along the relative position of ~250 cells. All cells were stained with HADA before imaging, and data are arranged such that the brighter HADA-stained pole was set to 0 on the *X*-axis—this is assumed to be the old pole. Normalized intensity values were calculated by dividing the mean intensity value of all cells at one position by the mean intensity value of all cells at all positions along the length of the cells. (**C**) Micrographs of the SepIVA-DAS L5::mCherry-FtsQ strain with stable SepIVA-DAS (top) and SepIVA-DAS destabilized by SspB expression (bottom). The scale bar is 5 µm and applies to all images. Arrowheads point to mCherry-FtsQ foci. (**D**) Percentage of total cells with FtsQ foci in SepIVA-stable (blue) and SepIVA-depleted (red) cells. A total of 253 cells across 3 biological replicates of depleted and 274 cells across 3 biological replicates of control strains were analyzed. ns, *P* > 0.05, calculated using an unpaired *t*-test. (**E**) Micrographs of SepIVA-DAS L5::mCherry-FtsW strain with stable SepIVA-DAS (top) and SepIVA-DAS destabilized by SspB expression (bottom). The scale bar is 5 µm and applies to all images. Arrowheads point to mCherry-FtsW foci. (**F**) Percentage of total cells with FtsW foci. A total of 300 cells across 3 biological replicates of depleted and 240 cells across 3 biological replicates of control strains were analyzed. ns, *P* > 0.05, calculated using an unpaired *t*-test.

Next, we sought to determine if SepIVA has a role in recruitment or stabilization of FtsQ or FtsW to the divisome. To test this, we used *M. smegmatis* SepIVA-FLAG-DAS strains expressing either mCherry-FtsQ or mCherry-FtsW. We previously validated the functionality of N-terminal fluorescent protein fusion to transmembrane divisome factors (32). We found that depletion of SepIVA did not affect the frequency of formation of FtsQ or FtsW foci (Fig. 2C through F). Our result indicating that SepIVA does not stabilize FtsQ at the divisome corroborates earlier work from the Nandicoori lab (41). Our data show that SepIVA is not a recruitment factor for FtsQ or FtsW. We have therefore excluded two of the three possible functions for non-enzymatic bacterial septum formation proteins—SepIVA is neither an FtsZ-anchoring protein (32) nor an assembly factor for the late divisome factors FtsQ and FtsW (Fig. 2). By process of elimination, we therefore hypothesized that it could have a role in allosteric activation of the divisome complex.

### SepIVA’s function in cell division is related to the activity of FtsW

To address whether SepIVA is helping activate the FtsWI complex, we hypothesized that FtsW gain of function could bypass SepIVA’s loss of function, as has been observed in other species for *ftsN* loss of function (10, 70). We previously described the *sepIVA* R234M mutant, which is specifically partially defective in the cell division function of SepIVA (40). We used *M. smegmatis* strains in which either wild-type (WT) *sepIVA* or the *sepIVA* R234M mutant was expressed from a single copy integrated at the L5 phage attachment site (L5::*sepIVA* WT or R234M) and introduced an anhydrotetracycline (ATc) inducible copy of *ftsW* (P*tet-FtsW),* to allow us to conditionally overexpress FtsW upon ATc treatment (Fig. S3A and B). The *sepIVA* R234M mutation slows division, resulting in longer cells as previously described (40) (Fig. 2A and B). Overexpression of FtsW also resulted in cells that are longer than WT, which we expect is due to excess protein causing inhibitory misregulation of the complex. When we overexpressed FtsW in the *sepIVA* R234M mutant, the cell division defect was suppressed, resulting in cell lengths comparable to WT (Fig. 3A and B). We also saw partial suppression of the growth defect when FtsW was overexpressed in *sepIVA* R234M mutants (Fig. S3C and E). To test if this suppression is specific to FtsW, we overexpressed FtsZ in the same strain backgrounds. Overexpression of FtsZ did not suppress either the cell length defect of the sepIVA R234M mutants (Fig. 3A and C) or the growth defect (Fig. S3D). We conclude from these results that SepIVA’s function is related to the activity of FtsW and that it is likely to be involved in allosteric activation of the divisome complex.

**Fig 3:**
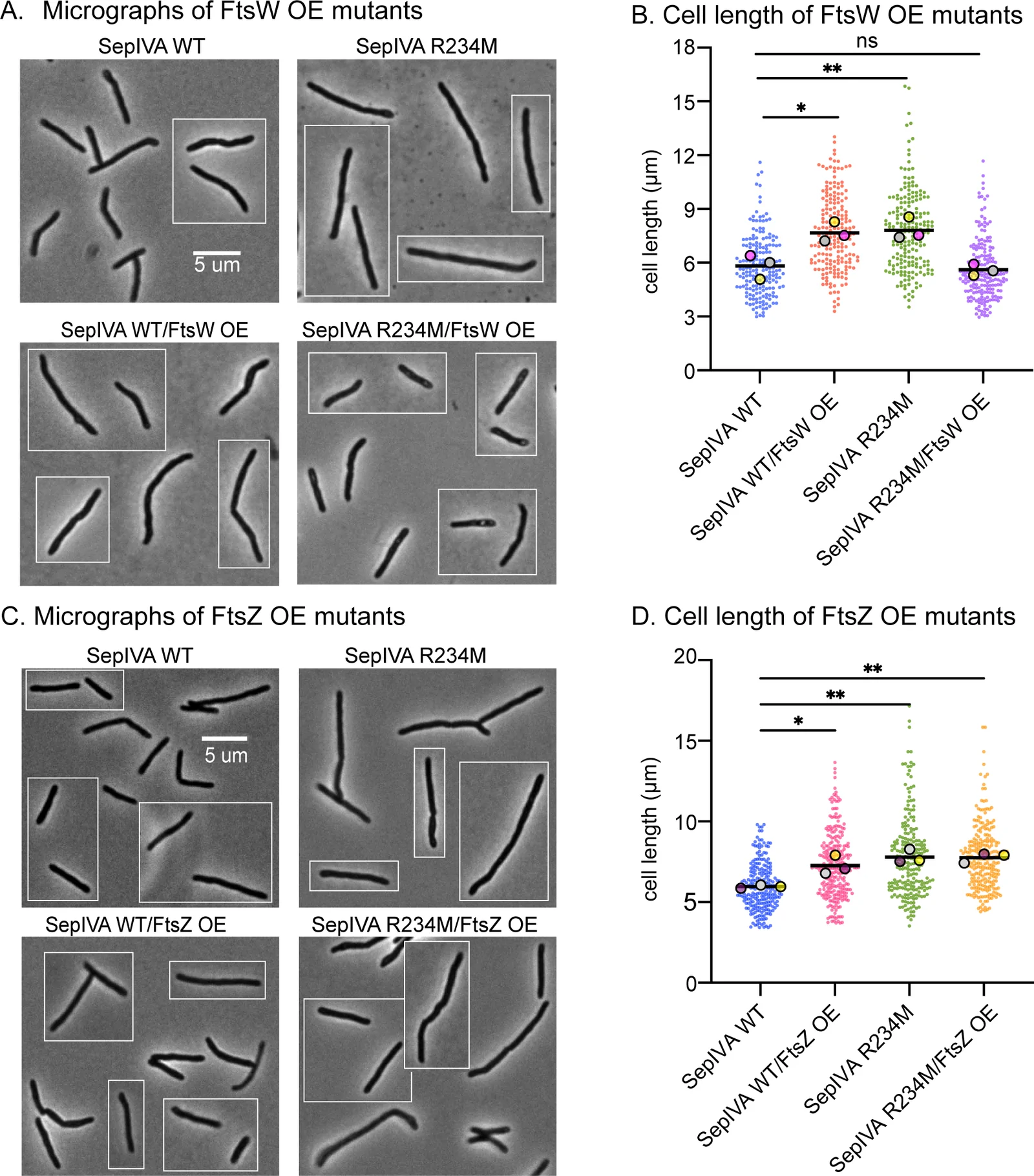
FtsW overexpression suppresses SepIVA’s cell division defects. (**A**) Micrographs of *M. smegmatis* cells expressing L5::*sepIVA* (WT or R234M) as the sole source of SepIVA, with or without P_tet_ FtsW, which causes FtsW overexpression—upon anhydrotetracycline (Atc) treatment. All strains were treated with (Atc) for 16 h. The scale bar of 5 µm applies to all images. (**B**) Quantified cell lengths of the strains from panel **A** for L5::*sepIVA* (WT or R234M) and Ptet FtsW mutants. (**C**) Micrographs of *M. smegmatis* cells expressing L5::*sepIVA* (WT or R234M) as the sole source of SepIVA, with or without P_tet_ FtsZ, which causes FtsZ overexpression—upon anhydrotetracycline (Atc) treatment. All strains were treated with (Atc) for 16 h. The scale bar of 5 µm applies to all images. (**D**) Quantified cell lengths of the strains from panel **A** for L5::*sepIVA* (WT or R234M) and Ptet FtsZ mutants. A total of 210 cells over 3 biological replicates for each strain were analyzed. Circles represent the mean of the individual replicates. *, *P* < 0.05; **, *P* < 0.01. Statistical differences between the mean values of the individual strains were determined using a two-way ANOVA.

### DNA damage does not impact septal localization of FtsZ, FtsQ, or FtsW

To better understand the regulation of divisome activation, we studied divisome proteins in a condition where the divisome should be inactive at synthesizing septal peptidoglycan. DNA damage inhibits cell division in bacteria (10, 13, 43, 71) and always acts at some point before activation of septal peptidoglycan synthesis so that the damaged chromosomes are not separated before they can be repaired (57, 72). To determine whether DNA damage inhibits septal association of conserved divisome proteins, we used merodiploid *M. smegmatis* strains expressing FtsZ-mCherry and GFP-FtsQ, or mCherry-FtsW. We grew the strains to log phase and then treated with mitomycin C (MitoC), which crosslinks DNA (73) and induces DNA damage responses (Fig. S4A). For the control, the cells were left untreated. We found that FtsZ still retained its original septal position despite MitoC treatment (Fig. 4A; Fig. S4B). Previous work suggested that ChiZ (Rv2719c, MSMEG_2742) inhibited cell division in DNA damage through inhibition of FtsZ assembly, though the effect of DNA damage alone on FtsZ assembly was never tested (57, 72). Our results suggest that ChiZ likely does not work through this mechanism, since FtsZ assembly is not inhibited in DNA damage (Fig. 4A and B; Fig. S4B). FtsQ foci were also present in most of the MitoC-treated cells. To determine if FtsQ foci were at the site of septation, we examined FtsQ co-localization with FtsZ, as FtsZ acts as a scaffold for FtsQ (21, 32). We saw that MitoC did not alter FtsQ-FtsZ co-localization (Fig. 4C). Similarly, FtsW foci were still seen in the cells upon treatment with MitoC (Fig. 4D), and there was no difference in their frequency between MitoC-treated and control cells (Fig. 4E). These data show that septal localization of FtsZ, FtsQ, or FtsW is not affected by DNA damage and suggest that the mycobacterial divisome is inhibited at a later step of divisome function during DNA damage.

**Fig 4:**
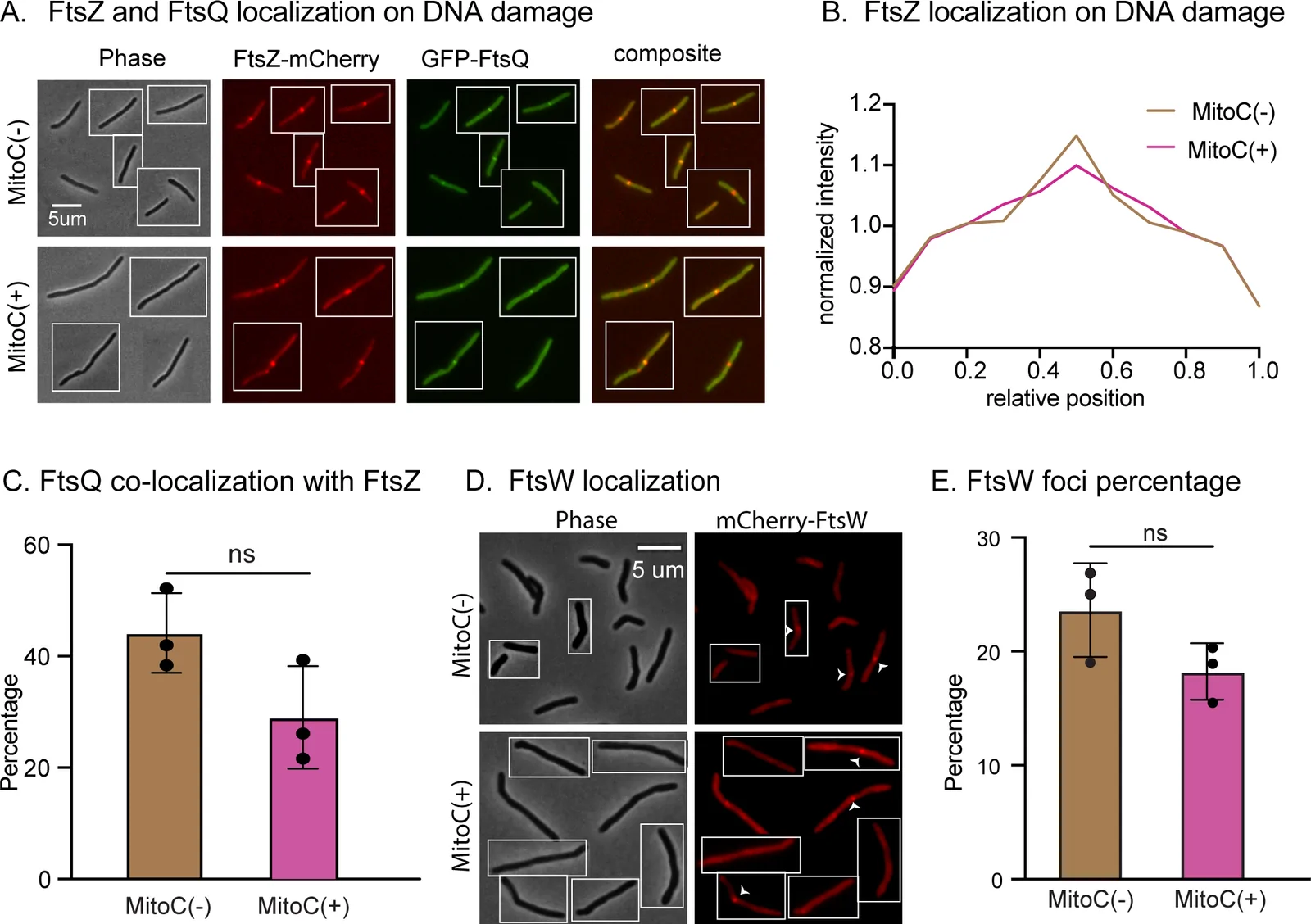
DNA damage does not impact septal localization of FtsZ, FtsQ, and FtsW. (**A**) Micrographs of *M. smegmatis* cells expressing FtsZ-mCherry and GFP-FtsQ. Scale bar is 5 µm and applies to all images. (**B**) Normalized mean intensity profiles of FtsZ-mCherry signal along the length of 260 cells (untreated) or 284 cells (MitoC-treated). Normalized intensity values were calculated by dividing the mean intensity value of all cells at one position by the mean intensity value of all cells at all positions along the length of the cells. (**C**) The percentage of cells exhibiting FtsQ-FtsZ focal co-localization among the cells with FtsZ foci. A total of 358 cells across 3 biological replicates in MitoC untreated condition and 458 cells across 3 biological replicates in MitoC-treated conditions were analyzed. (**D**) Micrographs of *M. smegmatis* cells expressing mCherry-FtsW in MitoC-untreated (top) and MitoC-treated (bottom). White arrowheads point toward the FtsW foci in the cells. Scale bar is 5 µm and applies to all images. (**E**) Percentage of mCherry-FtsW expressing cells that exhibit at least one FtsW focus under the two conditions. A total of 240 cells across 3 biological replicates of MitoC-untreated and 210 across 3 biological replicates of MitoC-treated cells were analyzed. ns, *P* > 0.05, calculated using unpaired *t*-tests.

### DNA damage inhibits septal localization of SepIVA

Next, we sought to determine if DNA damage impacts the localization of SepIVA. We treated a strain expressing GFP-SepIVA with MitoC or UV. UV causes DNA damage by creating pyrimidine dimers (74). For the controls, the strains were left untreated. We find that SepIVA is delocalized from the mid-cell in both types of DNA damage, while the sub-polar localization of SepIVA was unchanged (Fig. 5; Fig. S5E). The damaged cells were still alive (Fig. S5A and B). SepIVA delocalizes slowly, between 30 min and 2 h of MitoC (Fig. S5C and D). In conclusion, SepIVA is delocalized from the mid-cell in the presence of DNA damage, and this effect is due to the general response to DNA damage rather than a specific damage-inducing agent.

**Fig 5:**
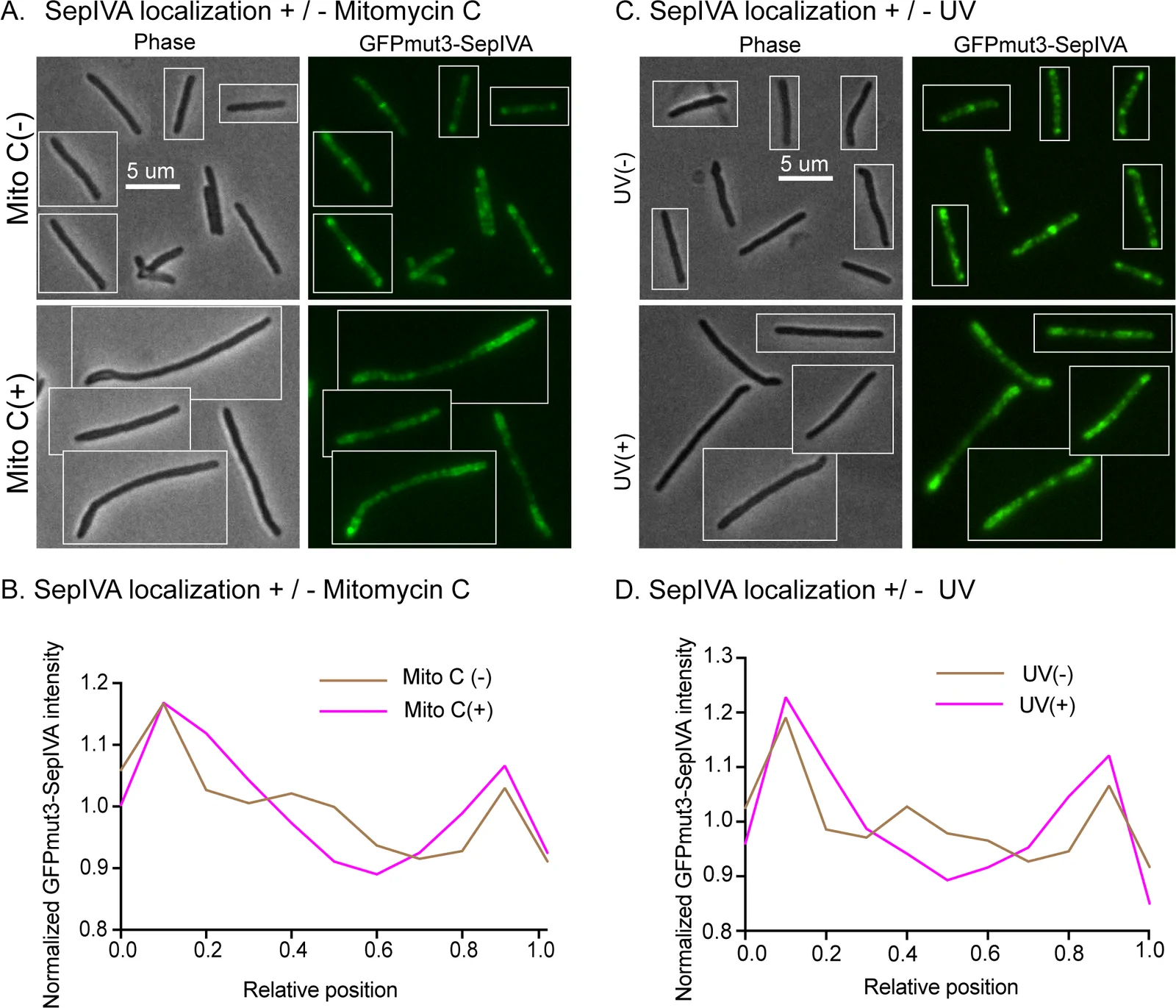
DNA damage inhibits septal localization of SepIVA. (**A**) Micrographs of *M. smegmatis* cells expressing GFP-SepIVA before and after 4.5 h of MitoC treatment at 350 ng/mL. Scale bar is 5 µm and applies to all images. (**B**) Normalized intensity profiles of GFP-SepIVA signal in untreated and mitoC-treated cells along the length of the cell, with the old pole set to 0 and the new pole set to 1 on the *X*-axis. A total of 310 cells for untreated and 340 cells for MitoC-treated, across 3 biological replicates, were analyzed. Normalized intensity values were calculated by dividing each position’s intensity value by the mean intensity value of GFP-SepIVA signal across the cell. (**C**) Micrographs of *M. smegmatis* cells expressing GFP-SepIVA before (top) and after (bottom) UV treatment of 15 mJ/cm^2^ and outgrown for 3 h. Scale bar is 5 µm and applies to all images. (**D**) Normalized intensity profiles of GFP-SepIVA signal before and after UV treatment, as in panel **B**. A total of 284 cells for MitoC-treated and 243 cells for untreated cells, across 3 biological replicates, were analyzed.

### DNA damage destabilizes the interaction between SepIVA and FtsQ

Next, we sought to determine if SepIVA and FtsQ’s interaction is disrupted in DNA damage, given that SepIVA delocalizes from the mid-cell while FtsQ persists (Fig. 4A and 5). We performed co-immunoprecipitations using an *M. smegmatis* strain expressing SepIVA-FLAG and FtsQ-strep as single copies and a strain expressing only SepIVA-FLAG as a negative control. Under standard growth conditions, SepIVA-FLAG efficiently co-purified when FtsQ-strep was pulled down with Strep-Tactin beads (Fig. 6), indicating a stable interaction between SepIVA and FtsQ even without cross-linking (32). We then investigated whether the interaction is maintained during DNA damage, and we chose UV -SepIVA delocalized in both UV and MitoC (Fig. 5). We observed a significant reduction in the co-immunoprecipitation efficiency of SepIVA-FLAG after UV-induced DNA damage compared with untreated controls (Fig. 6). We performed this experiment twice and got similar results (Fig. S6A) and quantified the efficiency of pulldown for each protein as a ratio of the elution over lysate band (Fig. 6B). The stability of both FtsQ-strep and SepIVA-FLAG remained unaffected by UV treatment, indicating that the decrease in SepIVA-FLAG recovery was due to a reduction of SepIVA-FtsQ association rather than a reduction in protein levels. These results demonstrate that while SepIVA and FtsQ are part of a stable complex during active growth, their interaction is reduced under conditions of DNA damage.

**Fig 6:**
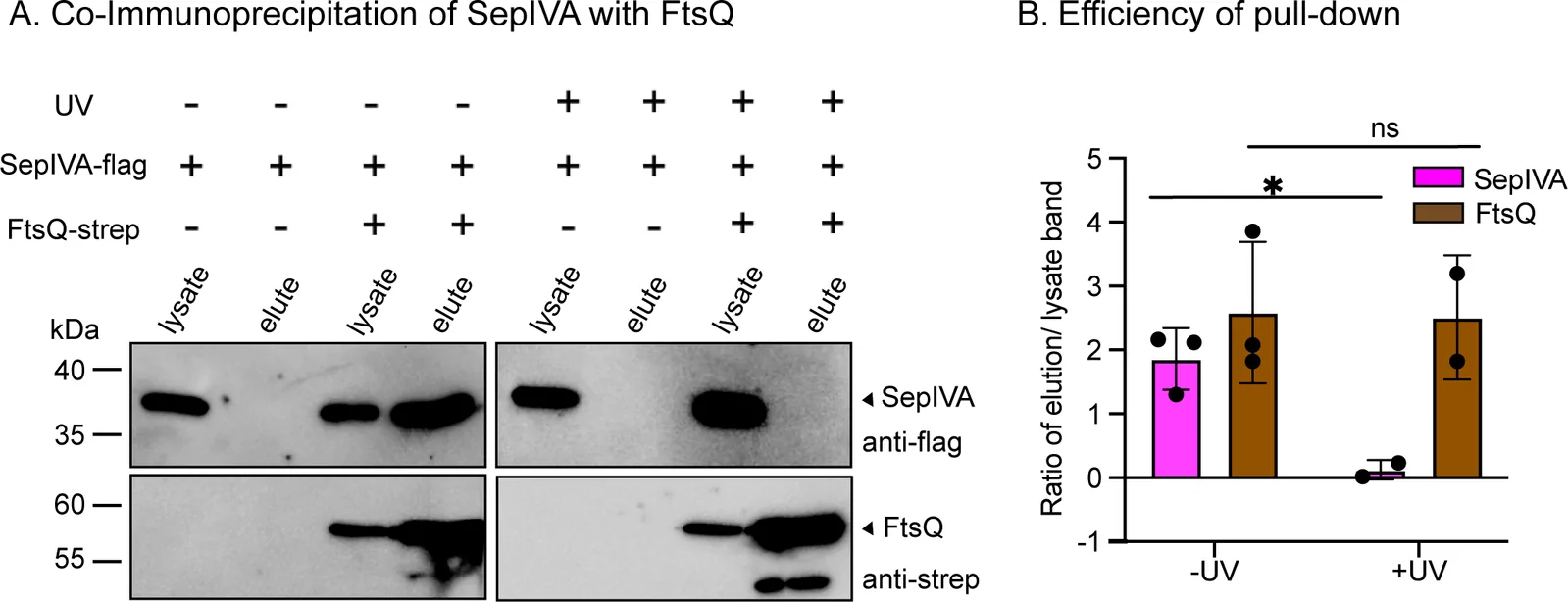
The interaction between FtsQ and SepIVA is destabilized during DNA damage. (**A**) α-FLAG and α-strep Western blots of lysate controls and immunoprecipitation elutions from the SepIVA-flag (control) and SepIVA-flag/FtsQ-strep (experimental) strains, using α-strep conjugated beads in the presence and absence of DNA damage (UV). The pulldown was performed twice, and a representative image is shown. (**B**) The efficiency of the immunoprecipitation (FtsQ) or the co-immunoprecipitation (SepIVA) is the ratio of the elution peak band intensity to the lysate band intensity for each experimental sample in panel **A**. The control samples, in which FtsQ was not strep-tagged, did not result in any apparent pulldown (left two lanes of each blot). Error bars represent standard deviation from two independent biological replicates. ns, non-significant (*P* > 0.05); *, *P* < 0.05, calculated using an unpaired *t*-test.

ChiZ has been identified as a potential regulator of cell division during DNA damage (57, 58). It is upregulated during DNA damage stress and interacts with FtsQ (58); thus, one model could be that ChiZ inhibits the interaction between SepIVA and FtsQ. However, we found that DNA damage still substantially inhibits cell division in an *Msmeg* ∆*chiZ* strain (Fig. S6B and C). Thus, if ChiZ does inhibit this interaction, it is not the only regulator involved.

## DISCUSSION

Our data support a model in which SepIVA’s association with the divisome contributes to the activation of the mycobacterial divisome complex. We speculate that SepIVA could help trigger an active conformation of the divisome through its interaction with FtsQ, resulting in activation of the FtsIW complex and initiation of peptidoglycan synthesis. This model is analogous to the well-characterized FtsQLB-FtsIW activation mechanism in *E. coli* (28, 30). This model is supported by our previous time-lapse microscopy showing that SepIVA is a late recruit to the divisome (32), like FtsN (32, 75, 76). Here, we show that it is recruited to the divisome complex by FtsQ but is not itself a recruitment factor for downstream factors like FtsQ or FtsW (Fig. 2). We were not able to test whether SepIVA recruits FtsI because we were unable to make a functional fusion. However, other work has shown that FtsI is recruited by FtsW (37, 77), and these proteins form a highly conserved protein complex, which usually works together (20, 78). Though we do not expect that SepIVA is a recruitment factor for FtsI, we have not eliminated the possibility that SepIVA could be recruiting FtsI or another undescribed divisome factor. The reduction in the HADA-stained division septa (Fig. 1) and maintenance of mCherry-FtsW foci (Fig. 2E and F) in SepIVA depletion suggest that FtsIW is not actively polymerizing even if it is physically present in the divisome. We hypothesize that the interaction between SepIVA and FtsQ could cause conformational rearrangements in the divisome complex that cue activation of the FtsIW enzymatic complex. The role of SepIVA in activation is supported by our data showing that in a *sepIVA* partial loss-of-function mutant (40), the diminished activation of FtsIW appears to be the rate-limiting step for septal peptidoglycan synthesis, since overexpression of FtsW in the *sepIVA* mutant strain suppresses the division defect (Fig. 3).

In *E. coli*, the recruitment of FtsN to the divisome complex signals the completion of divisome assembly. Its association cues conformational changes in FtsA and the FtsQLB complex that ultimately activate the FtsIW enzymes (27, 28, 69, 79, 80). Mycobacteria do not have *ftsN* or a *ftsA* homolog. FtsA is important for anchoring FtsZ to the membrane (81), in addition to its role in allosteric activation (27, 69, 82). The role of FtsA in anchoring the Z-ring is thought to be performed by SepF in mycobacteria (33). How the mycobacterial divisome is allosterically activated without either FtsN or FtsA has not been clear. However, other work has described direct interactions between FtsZ and FtsQ (38) and FtsZ and FtsW (37), which could help stabilize the FtsZ ring or could be involved in allosteric activation of the complex. We speculate that SepIVA may contribute to inducing a conformational rearrangement of the divisome, which could also involve these FtsZ-Q and FtsZ-W interactions.

FtsN is a transmembrane protein with a periplasmic peptidoglycan-binding domain (83), while SepIVA is strictly cytoplasmic. Our model is that SepIVA’s possible role in allosterically activating the divisome complex would be simpler than FtsN’s role in both activating and coordinating septation. Through its peptidoglycan-binding domain and transmembrane and cytoplasmic interactions with divisome factors, FtsN coordinates both septal synthesis and septal resolution, which occur nearly simultaneously in *E. coli* (84, 85). However, in mycobacteria, the entire septum is built before it is cleaved (61), and therefore, the mechanism to coordinate nearly simultaneous septal construction and resolution is not required. We propose that SepIVA may promote the active form of the divisome complex but that it is not involved in coordinating septal resolution. This likely works similarly in *B. subtilis* and other species where the full septum is built before being resolved (86). Indeed, time-lapse microscopy of divisome factors in *B. subtilis* indicates a simpler model for their motion dependent on septal peptidoglycan synthesis (87) than is seen in *E. coli* (85).

We found that the interaction between FtsQ and SepIVA, which we speculate could be important for activating septation, was inhibited during DNA damage (Fig. 6). The localization data support this, showing that FtsQ is at the divisome without SepIVA during DNA damage (Fig. 4 and 5). These results suggest that the interaction between FtsQ and SepIVA may be a key regulatory event during mycobacterial division. This could be altered by post-translational modification, or by other proteins. SepIVA is extensively arginine-methylated, and our previous studies indicate that these methylation sites regulate both elongation and division (40). FtsQ is phosphorylated, though its phosphorylation at threonine T24 (41, 88) does not affect its interaction with SepIVA (41); however, we have not yet tested whether DNA damage might activate other post-translational modifications. It might also be that a protein induced during DNA damage (53, 54) could block the interaction between FtsQ and SepIVA, analogous to similar inhibitory systems in *C. crescentus* (10) and *B. subtilis* (13); however, our results indicate that ChiZ does not fulfill this role (Fig. S5B and C).

## MATERIALS AND METHODS

### Bacterial strain and growth conditions

All *M. smegmatis* strains were cultured in 7H9 (Becton, Dickinson, Franklin Lakes, NJ, USA) medium supplemented with 5 g/L albumin, 2 g/L glucose, 0.85 g/L NaCl, 0.003 g/L catalase, 0.2% glycerol, and 0.05% Tween 80. Lysogeny broth Lennox plates were used for plating *M. smegmatis* and *E. coli* strains. Genetically unstable *M. smegmatis* strains, such as the *sepIVA* R234M strain, were cultured directly from plates immediately after construction. *E. coli* strains, Top10, XL1-Blue, and DH5α, were used for cloning. Antibiotic concentrations used for mycobacterial selection were kanamycin, 25 μg/mL; hygromycin, 50 μg/mL; nourseothricin, 20 μg/mL; and zeocin, 20 μg/mL. For *E. coli*, kanamycin, 50 μg/mL; nourseothricin, 40 μg/mL; and zeocin, 25 μg/mL were used. Anhydrotetracycline (aTc) was used at between 50 and 500 ng/mL for gene induction or repression.

### Strain construction

Plasmids were all made using Gibson cloning (89) (90). Vectors were sequence-confirmed and transformed into electrocompetent *M. smegmatis* strains. All strains used are listed in Table S1, plasmids used are listed in Table S2, and primers used are listed in Table S3. The SepIVA depletion strain was previously described (32). The strain with a knockout of *ftsQ* and a complementation of *ftsQ* at the L5 site was also previously described (32). We used L5 allele swapping to exchange plasmids at the L5 site of this strain (91). We used ORBIT recombineering as described (92) to integrate a vector that attaches a FLAG-DAS tag at the C-terminus of *sepIVA* in a strain that expresses FtsQ-strep from the L5 site. The ORBIT primer is in Table S3. We made the *chiZ* knockout using double-stranded recombineering as described (93).

### Cell staining

Cultures were grown to logarithmic phase and stained with 10 µM HADA (Tocris Bioscience) for 15 min with rolling and incubation at 37°C. HADA was then washed out, and the pellets were resuspended in 7H9 media. The cells were mounted on HDB agar pads before imaging. Images were either taken live or within 24 h of fixing them. Fixing of cells was performed by resuspending the pellets in phosphate-buffered saline (PBS)-Tween 80 with 1.6% paraformaldehyde solution for 15–20 min, followed by washing and resuspending them in PBS-Tween 80. The images were taken within 24 h of fixing.

### Microscopy and data analysis

Cells were immobilized on pads with 1.5% agarose in either HdB (for live-imaged samples) or PBS (for fixed samples) medium, and the images were taken on a Nikon Ti-2 widefield epifluorescence microscope with a Photometrics Prime 95B camera and a Plan Apo 100×, 1.45 numerical aperture lens objective. GFPmut3 fusions were imaged with a filter cube with a 470/40 nm excitation filter, a 525/50 nm emission filter, and a 495 nm dichroic mirror. mCherry2B fusions were detected using a filter cube with a 560/40 nm excitation filter, a 630/70 nm emission filter, and a 585 nm dichroic mirror. HADA was detected using a filter cube with a 350/50 nm excitation filter, a 460/50 nm emission filter, and a 400 nm dichroic mirror. All images were processed using NIS Elements software and analyzed using FIJI and MicrobeJ (94) to create cell ROIs and extract fluorescence data from them. Mean intensities, maximum intensities, and minimum intensities of at least 250 cells per genotype were quantified using MicrobeJ.

Mean intensity profiles were plotted using the “XStatProfile” plotting tool in MicrobeJ (94). *P*-values were calculated using ordinary one-way analysis of variance (ANOVA), Dunnett’s multiple comparisons test, with a single pooled variance using GraphPad Prism (v9.2). The mCherry-FtsQ foci were identified as foci that exceeded a threshold metric in which the ratio of the intensity of a focus over the mean intensity of a cell was greater than 1.65. The mCherry-FtsW foci in SepIVA depletion were identified as foci that exceeded a threshold metric in which the intensity of a focus over the mean intensity of a cell was greater than 1.55. The mCherry-FtsW foci in DNA damage were identified using a threshold metric in which the signal of a focus over the mean signal of a cell was greater than 1.44.

### Dependency of localization assays

For depletion of SepIVA and localization of FtsQ, 500 ng/mL anhydrotetracycline (Atc) was added to the SepIVA depletion strain (SepIVA-DAS) (CB 3290) in the early log phase and incubated by rolling at 37°C for 7–8 h, stained with HADA for 15 min followed by washing of HADA and fixing the cells. Localization of FtsW in the same background was performed similarly to FtsQ except that the cells were fixed for half an hour. The images were taken within 24 h of fixing. Similarly, for FtsQ depletion in *M. smegmatis* ∆*ftsQ* Ptet_OFF_::FtsQ (CB 3537), the cells were grown to log phase and treated with 500 ng/mL Atc for 7–8 h to deplete FtsQ transcriptionally and then stained with HADA for 15 min followed by washing with 7H9 media. The images were taken immediately after mounting, with no fixation.

### DNA damage assays

For the DNA damage assays, strains were treated with 350 ng/mL of mitomycin C in log phase and rolled for 5 h at 37°C. At 4 h and 45 min after mitoC treatment, 10 µM HADA was added, and the cells were rolled for an additional 15 min, before being washed in 7H9 media and mounted for imaging. For UV treatment of the cells, the strains were first grown to OD 0.3, poured into a clean petri dish, exposed to 15 mJ/cm^2^ UV in the UV crosslinker box (VWR), and then pipetted back into a culture tube. The culture was then incubated for 3 h by rolling at 37°C before imaging. For control, the cells were not exposed to UV.

### Co-immunoprecipitation

To test the interaction of SepIVA and FtsQ in normal and DNA-damaged conditions, 400 mL culture of the ftsQ-strep SepIVA-flag strain (CB 3785) and SepIVA-flag strain (CB 1160) strains were grown to late log phase (OD_600_0.6–0.9). One half of each culture was poured into 245 mm × 245 mm petri plates and exposed to 15 mJ/cm^2^ of UV light. The cultures were then pipetted back into a flask and incubated for 1 h 40 min with shaking at 37°C. Untreated cultures continued shaking at 37°C. The cultures were spun down and resuspended in wash buffer (100 mM Tris [pH 8.0], 150 mM NaCl, and 1 mM EDTA), mixed with protease inhibitor (Roche tablets and 1 mM phenylmethylsulfonyl fluoride), and 0.1% n-dodecyl-β-D-maltose (Cayman Chemical). The cells were lysed by being passed twice through a French press, and the lysate was centrifuged at 15,000 rpm for 15 min at 4°C. The protein concentration in the supernatant was normalized to 60 mg of total protein for all samples, which were incubated with 200 µL of Strep-Tactin-covered beads (IBA) for 2 h in the cold room. The beads were washed twice with wash buffer, and the protein was eluted with 40 µL of Buffer BX (IBA). Elutions were unfolded in Laemmli buffer and incubated at 95°C for 10 min before 15 µL of eluted samples were loaded onto an SDS gel. Thirty nanograms of total protein in lysate controls was also loaded. After SDS-PAGE, proteins were transferred onto polyvinylidene difluoride membranes. The membranes were probed with anti-flag (Sigma, F1804) antibody (1:10,000 dilution in Tris buffered saline, pH 8.0) and anti-strep (Invitrogen, MA5-44988) antibody (1:10,000 dilution in Tris buffered saline plus Tween 20 (TTBS)), which were then probed with anti-mouse secondary antibody (Invitrogen, 31430).

### RNA extraction and qRT-PCR

RNA extraction was done from three biological replicates of each strain. Twenty milliliters of each culture was grown for each strain to logarithmic phase (OD_600_ = 0.6–0.8). The cells were pelleted, resuspended in 750 µL of TRIzol reagent (Zymo Research), and lysed by bead beating. The RNA was purified using Zymogen Direct-zol RNA Miniprep Plus (catalog no. 2070) kit following the manufacturer’s protocol. One microgram of RNA from each sample was normalized and treated with dsDNase from Maxima H Minus cDNA Kit in a 10 µL reaction (Thermo, product no: M1681). The reverse transcriptase reaction was performed using Maxima H Minus cDNA Synthesis Master Mix. Complementary DNA was diluted to 100 ng/µL to use as a starting template for quantitative reverse transcription PCR (qRT-PCR). qRT-PCR was performed using SYBR Green qPCR Master Mix (Applied Biosystems, product number: A25742) using the manufacturer’s protocol. Primer3 was used to design the qPCR primers. SigA transcript levels were used as an internal control. The data were analyzed using the ΔΔCT method (95).

## SUPPLEMENTAL MATERIAL

Supplemental material mbio.01985-26-s0001.docx Supplemental figures and tables.

ASM does not own the copyrights to Supplemental Material that may be linked to, or accessed through, an article. The authors have granted ASM a non-exclusive, world-wide license to publish the Supplemental Material files. Please contact the corresponding author directly for reuse.
