# Supplementary material for: SepIVA has a function in activating the mycobacterial divisome, which is inhibited during DNA damage
Source: mBio. 2026 Sep 22;17(10):e01985-26. doi: 10.1128/mbio.01985-26 (PMC13712929; doi:10.1128/mbio.01985-26)
Supplement: Supplementary file 1 [file mbio.01985-26-s0001.docx]

**Supplemental figures:**

**Supplemental result associated with Figure 1:**

In Figure 1, we show that SepIVA is depleted in SepIVA-DAS strain, after induction of the SspB proteolytic adaptor. Here, we show the entire Western blot for the experiment (Fig. S1).

**Supplemental Figure 1: A.** α-FLAG Western blot of lysates from the SepIVA-DAS strain, without induction of the SspB proteolytic adaptor (control) and after 7-8 hours of induction (depleted). The samples were loaded onto the gel twice.

**Supplemental results associated with Figure 2.** Demographs of data in Figure 2AB.

**Supplemental Figure 2. Demographs of mCherry-SepIVA localization in control and *ftsQ*-depleted cells.** Additional analysis of images from Figure 2AB. Left: Control cells expressing FtsQ. Right: cells transcriptionally depleted for *ftsQ.* Cells are arranged by cell length, and mCherry-SepIVA signal is shown via the color scale on the right, with light colors indicating higher signal from the mCherry-SepIVA reporter.

**Supplemental results associated with Figure 3.** In this Figure, we show that overexpression of FtsW can suppress the cell division defects of a *sepIVA* R234M allele swap mutant. To determine whether Anhydrotetracycline (Atc) alone induced changes in cell length, we added Atc to both control strains that do not carry the P_Tet_ FtsW plasmid, and measured the cell lengths after overnight treatment. The result (Fig. S3A) shows that the cells lacking the Tet inducible promoter exhibited insignificant differences in cell length between ATc treated and untreated conditions. To confirm that the Ptet-FtsW plasmid successfully induced expression of *ftsW*, we performed QRT-PCR. The results (Fig. S3B) show that FtsW is induced in both strains after Atc treatment, and that *ftsW* levels are higher in the induced than in the control strains. To determine whether cell growth rates were also altered in the *sepIVA* R234M strain, and in the FtsW and FtsZ overexpression strains, we performed growth curves. The results (Fig. S3C) show that The *sepIVA* R234M strain grows significantly slower than the WT strain, but that FtsW overexpression alone does not impact bulk growth rates. When FtsW is overexpressed in the *sepIVA* R234M background, the slow growth defect is partially suppressed. However, when FtsZ is overexpressed, there is no suppression of the *sepIVA* R234M growth rate defect (Fig. S3D). Spot dilution assays show no viability phenotypes in any of these strains, though growth rate differences are also seen in colony size (Fig. S3E).

**Supplement figure 3**: **A.** Quantification of cell lengths of cells expressing L5:: *sepIVA* (WT or R234M) with or without P_Tet_ FtsW, which causes FtsW overexpression upon anhydrotetracycline (ATc) treatment. (+) denotes the strain treated with ATc , (-) denotes Atc untreated cells. ns, p>0.05 was calculated using an unpaired t-test. ****,p<0.0001. p value was calculated by using ordinary one way ANOVA. **B.** Relative expression of *ftsW* by qPCR on L5:: (*sepIVA* WT or *R234M*) with or without Ptet FtsW, following ATc treatment. Expression was normalized to *sigA*. **C.** Doubling time of L5:: *sepIVA* WT or L5::R234M with or without Ptet FtsW overexpression. **D.** Doubling time of L5:: *sepIVA* WT or L5::R234M with or without Ptet FtsZ overexpression. **E.** Spot dilution assay of L5:: *sepIVA* WT or L5::R234M with or without Ptet FtsW. ***, p<0.001, **, p<0.01 were calculated using an unpaired t-test.

**Supplemental results associated with Figure 4.** In Figure 4, we study how core essential divisome factors localize under DNA damage with MitoC. In these experiments, we used 350 ng/mL of MitoC for 5 hours before imaging. As these exact conditions have not been used before in assays with *M. smegmatis*, we sought to establish if the DNA damage response is activated in these conditions. We performed QRT-PCR on *Msmeg* treated with MitoC under these conditions to measure the level of transcription of genes known to be induced under DNA damage (1). We found that both *adnA*, a PafBC-induced gene, and *dnaE2*, an SOS-induced gene, are strongly induced under the conditions we used throughout the paper (Fig. S4A). These results indicate that both DNA damage responses are active under the conditions we tested. In Figure 4A,B, we describe how mCherry-FtsZ localization is impacted by DNA damage through MitoC. We show here the demographs of the data shown in Fig 4AB (Fig. S4B).

**Supplemental Figure 4: A.** Relative expression of the DNA damage-inducible genes *adnA* and *dnaE2* by qPCR in cells treated with mitomycinC (350 ng/ml) for 4 hours compared to untreated controls. Expression was normalized to *sigA*. **B.** Demographs of mCherry-FtsZ signal from images used for Figure 4AB. Cells are arranged by cell length, and mCherry-FtsZ signal is shown via the color scale on the right, with light colors indicating higher signal from the mCherry-FtsZ reporter.

**Supplemental Results associated with Figure 5.** In Figure 5, we show that SepIVA delocalizes from the divisome location after DNA damage with both MitoC and UV. To determine whether cells are alive after MitoC treatment, we assessed both recovery of HADA-labelled cell wall synthesis by microscopy and growth by growth curve analysis. We treated GFPmut3-SepIVA cells with mitomycin C (350 ng/ml) for 4 hours, washed and allowed recovery for 2, 4, 6, and 24 hours. We stained the cells with HADA to assess ongoing peptidoglycan synthesis as a measure of viability. The results (Fig. S5A) show that cells continued to incorporate HADA throughout the recovery period, indicating they remained metabolically active and did not die after treatment. Septum formation began to reappear at approximately 6 hours of recovery, and by 24 hours, septa had reformed in surviving cells, with SepIVA localization restored to a normal septal pattern. The growth curve shows that mitomycin C-treated cultures began to recover and resume growth approximately 14 hours after MitoC is washed out (Fig. S5B). Together, these results indicate that mitomycin C treatment does not kill the cells outright and cells are able to recover over time. To determine how quickly SepIVA localization changes under DNA damage, we performed microscopy on a GFPmut3-SepIVA strain treated with mitomycin C (350 ng/ml), examining cells at 0, 30 minutes, and 2 hours post-treatment. Delocalization of SepIVA from the septum occurred slowly, with no change at 30 minutes. By 2 hours, however, delocalization was clear (Fig. S5CD). In Figure S5E, we show demographs of the images shown and analyzed in Figure 5.

**Supplemental Figure 5:** **A.** Micrographs showing recovery of GFPmut3-SepIVA at 0, 2, 4 ,6 and 24 hrs of recovery after a 4 hr MitoC treatment and washout (washout is t=0). Top row: Phase contrast images, middle row: GFPmut3-SepIVA fluorescence, bottom row: HADA staining. Scale bar of 5 micron applies to all images. **B.** Growth curve of Gfpmut3-SepIVA cells under untreated, MitoC treated and post-treatment recovery conditions. **C.** Micrographs of GFPmut3-SepIVA treated with MitoC for 30 mins (middle), 2 hrs (bottom), and untreated(top). Scale bar of 5 micron applies to all images. **D.** Normalized intensity profiles of GFP-SepIVA signal in untreated and mitoC-treated cells (0, 2 hrs) along the length of the cell, with the old pole set to 0 and the new pole set to 1 on the X axis. Around 200 cells were analyzed for all untreated and MitoC treated, across two biological replicates. Normalized intensity values were calculated by dividing each position's intensity value by the mean intensity value of GFP-SepIVA signal across the cells. **E.** Demographs of imaging data from Figure 5. Cells are arranged by cell length, and mCherry-SepIVA signal is shown via the color scale on the right, with light colors indicating higher signal from the mCherry-SepIVA reporter.

**Supplemental Results associated with Figure 6.** In Figure 6, we show using co-immunoprecipitation that the interaction between SepIVA and FtsQ is weakened after DNA damage. Here, we show the entire Western blots for both replicate experiments (Fig. S6A). Previous work had associated *chiZ* (Rv2719c, MSMEG_2742) with a possible role in inhibiting cell division under DNA damage (2, 3); however, the requirement for *chiZ* to inhibit cell division under DNA damage was never directly tested. Here, we measure how DNA damage causes cell lengthening in the WT and ∆*chiZ* strains. We find that the ∆*chiZ* has only a modest defect in inhibiting cell division in MitoC treatment, suggesting that ChiZ plays a minimal role in this process and that there are likely other cell division inhibitors that are active under DNA damage (Fig. S6BC).

**Supplement figure 6**: **A**. α-FLAG and α-strep western blots of lysate inputs and immunoprecipitation elutions from the SepIVA-flag (control) and SepIVA-flag/ FtsQ-strep (experimental) strains. Protein was collected from untreated and UV-treated cells, then complexes were captured using StrepTactin conjugated beads. The co-immunoprecipitation was performed twice, and whole-blot images from both trials (replicate 1 and replicate 2) are shown. **B.** Micrographs of WT *Msmeg* and ∆*chiZ* strains treated with Mitomycin C for 4 hours at 350 ng/mL (bottom) and untreated (top) **C**. Cell lengths of the strains from panel B. Bars represent the mean. 220 cells over three biological replicates for each strain were analyzed. ****, p<0.001 were calculated using an unpaired t-test.

**Supplemental table 1(Table S1): Strains**

| Strain# | Nickname | genotype | Figure Panel |
| --- | --- | --- | --- |
| CB1166 | *sepIVA*-DAS | mc^2^155 MSMEG_2416::FLAG-das-P38-orbit gi::pGMCgS-TetOFF-18-sspB | 1 |
| CB3537 | Ptet_OFF_::*ftsQ,* mCherry-SepIVA | mc^2^155 ∆ftsQ::zeoR L5:: pMC1s-ptetOFF4-FtsQ giles:: pCT267-mcherry-SepIVA clone2 | 2a,b |
| CB3290 | *sepIVA*-DAS,mCherry-FtsQ | mc^2^155 MSMEG_2416::FLAG-das-P38-orbit L5::AN003-TetON-mcherry-FtsQ:: pGMCgS-TetOFF-18 sspB | 2c,d |
| CB3652 | *sepIVA*-DAS, mCherry-FtsW | mc^2^155 MSMEG_2416::FLAG-das-P38-orbit tw:: Ptb21-mCherry-FtsW:: pGMCgS-TetOFF-18 sspB | 2e,f |
| CB1204 | L5:: *sepIVA* WT | mc^2^155 ∆sepIVA::zeoR L5::pKK158-PsepIVA-sepIVA | 3 |
| CB3808 | N/A | mc^2^155 ∆sepIVA::zeoR L5::pKK158-PsepIVA-sepIVA/ Ct-267-FtsW-tetR | 3 |
| CB3809 | N/A | mc^2^155 ∆sepIVA::zeoR L5::pKK158-PsepIVA-sepIVA/Ct-267-RBSmut_FtsW-tetR | 3 |
| CB3821 | L5:: *sepIVA* WT | mc^2^155 ∆sepIVA::zeoR L5:: pKK216 - PsepIVA-SepIVA-strep | 3 |
| CB3822 | L5:: *sepIVA* R234M | mc^2^155 ∆sepIVA::zeoR L5:: pCT94-sepIVA-strep (R234M) | 3 |
| CB3823 | L5:: *sepIVA* WT, pTet-*ftsW* | mc^2^155 ∆sepIVA::zeoR L5:: pKK216 - PsepIVA-SepIVA-strep /Ct-267-RBSmut_FtsW-tetR | 3 |
| CB3824 | L5:: *sepIVA* R234M, pTet-*ftsW* | mc^2^155 ∆sepIVA::zeoR L5:: pCT94-sepIVA-strep (R234M) /Ct-267-RBSmut_FtsW-tetR | 3 |
| CB858 | L5::GFP-*ftsQ* tweety:: *ftsZ*- mCherry | mc^2^155 ∆ftsQ::zeoR L5::KK216 - mGFPmut3-ftsQstrep tweety:: pMCtH - p21 - ftsZ(wk) - mCherry2B | 4a,b,c |
| CB3635 | mCherry-*ftsW* | mc^2^155/ ptw-Ptb21-mCherry-FtsW clone2 | 4d,e |
| CB1699 | GFP-*sepIVA* | mc^2^155 L5::pCB910-gfp-sepIVA | 5 |
| CB3785 | SepIVA-flag/ *ftsQ*-strep | mc^2^155 ∆ftsQ::zeoR L5::ct16_pUV15(w/o tetO)_FtsQ-strep MSMEG_2416::FLAG-das-P38-orbit | 6 |
| CB1160 | *sepIVA*-flag | mc^2^155 MSMEG_2416::FLAG-das-P38-orbit | 6 |
| CB3312 | ∆*chiZ* | mc^2^155 ∆*chiZ*::zeoR | S5bc |

**Supplemental Table 2 (Table S2): Plasmids**

| (CB number) | description | Used in strains | Reference for parent vector | Remarks |
| --- | --- | --- | --- | --- |
| 3534 | Top10/ pCT267-mcherry-SepIVA Giles(hyg) | 3537 | This work |  |
| 2867 | Top10/ pMC1s-ptetOFF4-FtsQ | 3537 | This work |  |
| 3165 | top10/AN003-pL5-mcherry-FtsQ | 3290 | This work |  |
| 3633 | Top10/ptw-Ptb21-mCherry-FtsW | 3562, 3635 | This work | Synthesized by Azenta |
| 1036 | pGMCgS-TetOFF-18 sspB | 1166,  3290, 3652 | (4) |  |
| 1694 | DH5a / pCB910-gfp-sepIVA | 1699 | (5) |  |
| 3749 | Top10/Ct16-PUV(w/o TetO)_FtsQ-strep | 3785 | This work |  |
| 1052 | DH5a/pKM461 | 3785 | (6) |  |
| 3784 | Top10/Ct-267-FtsW-tetR | 3808 | This work |  |
| 3807 | Xl1-B/Ct-267-RBSmut_FtsW-tetR | 3809, 3823, 3824 | This work |  |
| 2556 | Top10/pCT94-sepIVA-strep (R234M) |  | (7) |  |
| 1213 | DH5a / pKK216 - PsepIVA-SepIVA-strep |  | (7) |  |

**Supplemental Table 3 (Table S3): Primers**

| Plasmid/  strain | Feature | Primers |
| --- | --- | --- |
| CB2867 | pMC1s-ptetOFF4-FtsQ | Accacccagctttcttgtacaaagtggctctccatcgatgtgaccgggacgggtcccca  cccttttttattttatccatggatccagctgcagaattcctatttgaccgtcggcaggt |
| CB 3534 | pCT267-mcherry-SepIVA | ccacggatccgcatgcttaattaagaaggagaTCACATATGGCCAGCGATAGCACTGAG  ccgcggcgcagccgggtgaggatgctgcccacAGCGCTACTGGATCCGCTAGATCCCTG |
| CB3165 | pL5-mcherry-FtsQ | CGATCCGCATGTTAATTAAGAAGGAGATATACATatgGATAGCACTGAGAGCGGCTCCA  gcggggtcaccgtggggacccgtcccggtcacGGTACCACTGGATCCGCTAGATCCCT |
| 3749 | Ct16-PUV(w/o TetO)_FtsQ-strep | TTTATTTGATGCCTGGCAGTCGATCGTACGCTAGTTAACCACTCTAGAAATATTGGATC  gacccgtcccggtCATCGATATATCTCCTTCTTAATTAACATGCGGATCGTGCTCATTT  tcttcagcggggtcaccgtggggacccgtcccggtCATCGATATATCTCCTTCTTAATT |
| 3784 | Ct-267-FtsW-tetR | cgatccgcatgcttaattaagaaggagatataACTAGTatgggcagcatcctcacccg  GCGGCCGCGGTACCAGATCTTTaacACAACGTCGTGACTGGGAAAACCCTGGCGTTACC  GGTAACGCCAGGGTTTTCCCAGTCACGACGTTGTgttAAAGATCTGGTACCGCGGCCGC  cgccaagctctaatacgactcactatagggaagctagttTGATTCTGTGGATAACCGTA |
| 3807 | Ct-267-RBSmut_FtsW-tetR | catttcgacgccgagagattcgccgcccgaaatgagcacgatccgcatgcttaattaagatcgagatataACTAGTatgggcagcatcctcacccggctgcgccgcggcggcgccccgggtgacg |
| 3785 | SepIVA-FLAG_P38_orbit | accgcaatcctacggcgacgccatattcgcgccgcggcacgtcgccgtcaccgcaggttgagcag atgcGGTTTGTACCGTACACCACTGAGACCGCGGTGGTTGACCAGAC AAACCgcgggtcacgtagtcgtgtgtccccgcggtggtccgcaactggtgacgtccgcggccgac cgaccgcagg |
| 3312 | mc^2^155 ∆*chiZ*::zeoR | Gatacgccccagaaccgg  Tgtagagcttctttcctgcact  cgaggagcaggacTGAATAACT  gactatctgagccgggatctg |
| qRTPCR | FtsW F | agatgctgatcccgctgg |
| qRTPCR | FtsW R | cagggatgacacgaagacct |
| qRTPCR | *dnaE2* F | gcactggcacatcctcac |
| qRTPCR | *dnaE2* R | ccgaaccggtccactagat |
| qRTPCR | *adnA* F | CAG GAC CGA ACC CGA AAC |
| qRTPCR | *adnA* R | CAC AGA CCT TCC TGG ACA C |
| qRTPCR | *sigA* F | GACTACACCAAGGGCTACAAG |
| qRTPCR | *sigA* R | TTGATCACCTCGACCATGTG |
|  |  |  |

**References:**

1.	Adefisayo OO, Dupuy P, Nautiyal A, Bean JM, Glickman MS. 2021. Division of labor between SOS and PafBC in mycobacterial DNA repair and mutagenesis. Nucleic Acids Research 49:12805–12819.

2.	Chauhan A, Lofton H, Maloney E, Moore J, Fol M, Madiraju MVVS, Rajagopalan M. 2006. Interference of Mycobacterium tuberculosis cell division by Rv2719c, a cell wall hydrolase. Molecular Microbiology 62:132–147.

3.	Vadrevu IS, Lofton H, Sarva K, Blasczyk E, Plocinska R, Chinnaswamy J, Madiraju M, Rajagopalan M. 2011. ChiZ levels modulate cell division process in mycobacteria. Tuberculosis (Edinb) 91:S128–S135.

4.	Kim J-H, Wei J-R, Wallach JB, Robbins RS, Rubin EJ, Schnappinger D. 2011. Protein inactivation in mycobacteria by controlled proteolysis and its application to deplete the beta subunit of RNA polymerase. Nucleic Acids Res 39:2210–2220.

5.	Wu KJ, Zhang J, Baranowski C, Leung V, Rego EH, Morita YS, Rubin EJ, Boutte CC. 2018. Characterization of Conserved and Novel Septal Factors in *Mycobacterium smegmatis*. Journal of Bacteriology 200:e00649-17, /jb/200/6/e00649-17.atom.

6.	Murphy KC, Nelson SJ, Nambi S, Papavinasasundaram K, Baer CE, Sassetti CM. 2018. ORBIT: a New Paradigm for Genetic Engineering of Mycobacterial Chromosomes. mBio 9:e01467-18.

7.	Freeman AH, Tembiwa K, Brenner JR, Chase MR, Fortune SM, Morita YS, Boutte CC. 2023. Arginine methylation sites on SepIVA help balance elongation and septation in *Mycobacterium smegmatis*. Molecular Microbiology 119:208–223.
